# From reproductive violence to empowerment: reconquering power in assisted reproduction – a qualitative study

**DOI:** 10.1080/26410397.2026.2694254

**Published:** 2026-07-03

**Authors:** Virginie Rozée, Hélène Malmanche, Elise de La Rochebrochard

**Affiliations:** a Senior Researcher, Sexual and Reproductive Health and Rights Research Unit, UR14, Institut National d'Etudes Démographiques (INED), Aubervilliers, France.; b Postdoctoral Researcher, Senior Researcher, Sexual and Reproductive Health and Rights Research Unit, UR14, Institut National d'Etudes Démographiques (INED), Aubervilliers, France; c Senior Researcher, Sexual and Reproductive Health and Rights Research Unit, UR14, Institut National d'Etudes Démographiques (INED), Aubervilliers, France; Université Paris-Saclay, Univ. Paris-Sud, UVSQ, CESP, INSERM, Villejuif, France

**Keywords:** Reproductive violence, gender-based violence, gynaecological violence, cross-border reproductive care, assisted reproductive technology

## Abstract

People who use medically assisted reproduction (MAR) outside the legal and/or medical framework are generally on the margins of the dominant procreative norm, making them particularly vulnerable to a wide range of reproductive violence such as legal restriction to medical care, discrimination or mistreatment. The aim of this study was to explore experiences of reproductive violence among people living in France who sought MAR either abroad or without medical assistance. Semi-structured interviews were conducted between 2022 and 2023. Almost half of the interviewees (n = 28/69) had been confronted with reproductive violence during their MAR journey. Although the French bioethics law extended access to MAR to single women and female same-sex couples in 2021, participants described how the shortage of gametes fuelled all forms of reproductive violence, encompassing psychological, economic and physical violence. Our findings revealed an intersectional dimension to these experiences, particularly affecting individuals with non-heterosexual identities, older age or higher body weight. Single women appeared particularly exposed to such violence. However, faced with reproductive violence, many participants demonstrated resilience and empowerment, notably through support from social networks and associations. This solidarity helped them to develop strategies of resistance and avoidance. From personal difficulties and experiences of violence at the institutional level and in care, people moved on to awareness of systemic violence, encouraging them to support their peers. Awareness led them to denounce reproductive injustice and even to demand changes in MAR organisation and care. *DOI: 10.1080/26410397.2026.2694254*

## Plain language summary

This article looks at the difficulties some people face when trying to have a child through fertility treatment. Some of these difficulties are forms of “reproductive violence.” This means harms caused by not respecting people's right to make their own decisions about reproduction. Most research on reproductive violence focuses on abortion or childbirth. We looked at whether it occurs when people have fertility treatment, which constitutes a marginal way to become parents. We interviewed 69 people in 2022–2023 who could not get treatment in the French healthcare system because of legal restrictions. The majority had travelled to neighbouring countries. In 2021, France changed its law to give all women access to fertility treatments, regardless of their relationship status. But even one year after the law had changed, challenges continued. Our study shows that people are often judged by healthcare professionals and not always treated with respect. Instances of reproductive violence when seeking MAR often involve people who do not fit the common image of the “ideal parent.” They might be single women or aged over 40 or have higher body weight. Overloaded medical centres and a shortage of donors make access even harder, driving many women to seek care abroad. Despite these challenges, many found support through online communities and associations. These groups enable women to better prepare themselves, avoid mistreatment and become more aware of their rights. Negative experiences have led many people to begin demanding more equal and respectful infertility care.

## Résumé

Les personnes ayant recours à la procréation médicalement assistée (PMA) en dehors du cadre médical et/ou légal se situent généralement en marge de la norme procréative dominante, ce qui les rend particulièrement vulnérables à diverses formes de violence reproductive comme les restrictions juridiques d’accès aux soins, la discrimination ou les mauvais traitements. Cette étude avait pour objectif d’explorer les expériences de violence reproductive chez les personnes vivant en France qui avaient demandé une PMA à l’étranger ou sans assistance médicale. Des entretiens semi-structurés ont été menés entre 2022 et 2023. Près de la moitié des personnes interrogées (n=28/69) avaient été confrontées à des violences reproductives pendant leur parcours de PMA. Même si la loi française sur la bioéthique a étendu l’accès à la PMA aux femmes célibataires et aux couples de femmes en 2021, les participantes ont décrit comment la pénurie de gamètes alimentait toutes les formes de violence reproductive, englobant les violences psychologiques, économiques et physiques. Nos résultats ont révélé une dimension intersectionnelle de ces expériences, affectant en particulier les personnes ayant une identité non hétérosexuelle, un âge plus avancé ou un poids corporel plus élevé. Les femmes célibataires semblaient particulièrement exposées à cette violence. Néanmoins, face aux violences reproductives, beaucoup de participantes ont fait preuve de résilience et d’autonomisation, notamment grâce au soutien des réseaux sociaux et d’associations. Cette solidarité les a aidées à élaborer des stratégies de résistance et d’évitement. Ayant rencontré des difficultés personnelles et des expériences de la violence au niveau institutionnel et dans les services de soins, les personnes ont pris conscience des violences systémiques, ce qui les a encouragées à soutenir leurs pairs. Cette sensibilisation les a incitées à dénoncer les injustices reproductives et même à exiger des changements dans l’organisation et les soins liés à la PMA.

## Resumen

Las personas que utilizan las técnicas de reproducción asistida (TRA) fuera del marco legislativo y/o médico generalmente están al margen de la norma procreativa dominante, por lo que son particularmente vulnerables a una amplia gama de violencia reproductiva, tales como restricción legislativa a la atención médica, discriminación o maltrato. El objetivo de este estudio era examinar las experiencias de violencia reproductiva de personas que viven en Francia que buscaron TRA, ya sea en el extranjero o sin asistencia médica. Entre 2022 y 2023, se realizaron entrevistas semiestructuradas. Casi la mitad de las entrevistadas (n = 28/69) habían enfrentado violencia reproductiva durante su trayectoria de TRA. A pesar de que la Ley de Bioética de Francia de 2021 extendió el acceso a TRA a mujeres solteras y parejas de lesbianas, las participantes describieron cómo la escasez de gametos alimentó todas las formas de violencia reproductiva, entre ellas violencia psicológica, económica y física. Nuestros hallazgos revelaron la dimensión interseccional de estas experiencias, que afecta en particular a las personas con identidades no heterosexuales, de edad más avanzada o con sobrepeso. Las mujeres solteras parecían ser especialmente vulnerables a tal violencia. Sin embargo, al enfrentar violencia reproductiva, muchas de las participantes demostraron resiliencia y empoderamiento, notablemente gracias al apoyo brindado por redes y asociaciones sociales. Esta solidaridad las ayudó a formular estrategias de resistencia y evitación. A raíz de dificultades y experiencias personales de violencia a nivel institucional y en la atención médica, las personas pasaron a adquirir conciencia de la violencia sistémica, lo que las motivó a apoyar a sus pares y las llevó a denunciar la injustica reproductiva e incluso a exigir cambios en la organización y los servicios de TRA.

## Introduction

Reproductive violence encompasses all infringements to reproductive integrity and autonomy – essentially, all failures to uphold reproductive rights.^1^ It has been analysed in feminist studies since the 2000s and gained greater attention in the 2010s,^2^ in particular with the development of work on reproductive justice.^3^ It occurs across a variety of areas related to procreation and non-procreation, such as abortion, contraception and childbirth, among others. Reproductive violence broadens the scope of gynaecological and obstetric violence, which has been the subject of a growing number of feminist studies^4–6^ and institutional reports.^7,8^ In 2021, Chadwick and Mavuso defined reproductive violence as “practices, representations, policy, state, and institutional efforts to coerce, control, punish, diminish, devalue or oppress the reproductive capacities/bodies of marginalised peoples.”^2^ This violence targets the reproductive body in its personal, social and political dimensions.^9^ The term “violence” highlights the structural dimension of these experiences,^10^ integral to the patriarchal and paternalistic history of reproductive medicine and the continuum of gender violence.^11^ As a gender-based violence, reproductive violence is structural^12^ and may be “indirect, anonymous and invisible, and produced by the State and its institutions, or by social norms that reproduce social inequalities.”^13^

Moreover, intersectional feminism has shed light on the cumulative effects of discrimination on people who belong to several minorities and oppressed groups, such as gender minorities including LGBTQIA+, racialised people,* especially those socially perceived as non-white in Western societies, people with disabilities and poor people.^14,15^ The conjunction of reproductive violence and intersectional feminism led scholars to shed light on the stratification of reproduction:^16,17^ some reproductive projects, choices or bodies are promoted and valued, while others are prevented or devalued. In this context, public policies, laws, medical care and more generally normative injunctions may sustain and accentuate reproductive injustice.^18^

Research on reproductive violence has shed new light on discriminatory social practices in the fields of childbirth, birth control and sexuality.^19^ Striking examples of reproductive violence have emerged, such as forced medical sterilisation in the 1960s and 1980s, particularly among indigenous women in Latin America, poor men from rural areas in India, HIV-positive women in South Africa and among people with disabilities and poor, racialised women in French overseas territories.^20^

Conversely, the conceptual framework of reproductive violence has not yet been applied to the field of medically assisted reproduction (MAR). Reproduction through MAR (including artificial insemination and *in vitro* fertilisation techniques) is a minority practice. In France, for example, it accounted for 3.7% of births in 2022,^21^ although it has constantly increased over the last decades.^22^ From a normative point of view, using MAR is still a deviation from the dominant social norm for making a family, i.e., the heterosexual couple that spontaneously conceives a child.^23^ The literature shows that individuals who access MAR, in their home country or abroad, are generally socioeconomically privileged, given that these techniques most often develop within a private, and therefore costly, market.^24,25^ But MAR is also used by people with fewer socioeconomic resources, including those who cross borders to obtain reproductive care.^26^ The vulnerability captured by the concept of reproductive violence cannot be reduced to educational attainment or economic resources alone. The few studies in the social sciences on access to MAR for racialised, poor or overweight or obese people^27–29^ shed direct light on certain categories of violence within MAR. Such violence may intensify towards individuals who are more marginalised and vulnerable, in particular those who use assisted reproduction outside their national legal and/or medical framework. As they represent a very small proportion of the people who use MAR, themselves already a minority and “on the margins” of social norms, people who travel to obtain cross-border reproductive care may be seen as even more marginal, as being “on the margins of the margins”. Given this status, they may be labelled “deviant”.^30^ As such, they are particularly exposed to discrimination and stigmatisation through institutional, interpersonal, psychological, physical and social violence.

France is an interesting terrain for examining reproductive violence in MAR. It has one of the most accessible and supportive healthcare policies in the world regarding financial coverage for MAR. Since the first French bioethics law of 1994, which governed the organisation and conditions of access to MAR in the country, all legal treatments are fully reimbursed, though subject to a certain age limit and a certain number of attempts. This law structured the French “bioethical model,”^31^ based, on the one hand, on prioritising access to MAR within the public healthcare system (in line with a universal approach to healthcare access), and, on the other hand, on rejecting any financial transaction involving the human body (gamete donation is strictly non-remunerated for donors and free of charge for recipients).

In 2021, after lengthy and intense debates within Parliament and civil society, the bioethics law^†^ has been revised to be more inclusive.^32^ Before revision, French legislation on access to MAR was one of the most restrictive in Europe.^33^ Certain techniques such as social egg freezing were prohibited, while access to authorised techniques was restricted to heterosexual couples “of childbearing age”. Since 2021, sperm donation is authorised for single women and female couples, who are now permitted to receive fertility treatments which are fully covered and reimbursed. However, many inequalities and prohibitions remain, showing differentiated access to MAR.^34^ Gay couples and trans persons are not included in the law regulating access to MAR techniques, while surrogacy, preimplantation genetic diagnosis (PGD-A) (genetic screening of embryos to reduce the risk of miscarriage) and the importation of gametes from abroad are still prohibited.^32^

In this article, our aim is to analyse MAR practices from the perspective of reproductive violence by interviewing people living in France and using MAR outside the French legal and/or medical framework. This project was developed with the participation of associations that help French people with procedures such as cross-border reproductive care or “at- home” insemination.

## Materials and methods

The research project “Outside-ART”^‡^ on which this article is based aims to explore MAR among people living in France, with a specific focus on MAR used outside the French legal and/or medical framework, by means of a quantitative online survey and a subsequent qualitative study.

### Study design

The present study is based on interviews with people living in France who used assisted reproduction through cross-border reproductive care or in a non-medical setting, before and/or after the new French bioethics law.

### Recruitment of participants

The participants in the qualitative study were recruited from the quantitative survey aiming to collect data on the profile, motivation and experience of MAR users in France and outside the French legal and/or medical framework (countries, techniques, etc). The online survey was developed and hosted by GIDE, a web survey provider, using their SCROLL® platform. It was carried out between October 2021 and September 2023. At the end of the questionnaire, respondents were asked if they would agree to take part in a semi-structured interview and, if so, to provide their contact details.

Participants in the online survey were recruited through an extensive advertising campaign on social media and through the network of associations partnering with the project. Invitations to complete the online questionnaire targeted people who had experience of MAR performed in France, abroad or at home, whether they had used MAR in the past, were currently using it or simply considering it.

### Selection criteria

The selection criteria for the qualitative study were based on information collected in the online survey: (1) age at least 18 at the time of completing the online questionnaire (a mandatory requirement for access), (2) completion of the questionnaire between October 2021 and May 2022 (due to scheduling constraints on the qualitative fieldwork, participants who completed the questionnaire between June 2022 and September 2023 were not considered for the qualitative study), (3) written agreement to take part in a semi-structured interview following completion, (4) provision of usable contact details, (5) declaration that the respondent was undergoing or had already undergone MAR abroad while living in France, had imported sperm from abroad or had made a private arrangement at home and (6) had undergone MAR before and/or after the revision of the law in France. Among all respondents to the online survey during this period, three hundred respondents met these selection criteria. All of them were contacted and invited for an interview. Sixty-nine of them (23%) participated in the qualitative study.

### Data collection

Interviews were carried out between June 2022 and January 2023. The semi-structured interviews began with an opening question: what can you tell us about your MAR journey? This was followed by a flexible interview guide, which had been tested beforehand and covered the following topics: the MAR process, choices and decision-making, the organisation of the process and the difficulties encountered, if any, the respondent’s experience, opinion and representations of MAR (see Supplementary Material). We planned to approach gynaecological violence, a topical and controversial issue in France, at the end of the interviews. However, most interviewees spontaneously raised this issue.

Interviews were conducted in French by telephone, videoconference or face-to-face, depending on the interviewee's preference. They lasted between 25 minutes and 2 hours, with a median duration of 55 minutes. They were carried out by one or other of the first two authors (VR and HM), who are white cisgender female researchers who have been working in the field of reproductive health and rights for several years, one of them (HM) being a midwife with training in infertility care and support skills for women and men undergoing MAR. Most of the interviewees were alone during the interview; nine were with their young child, and two had their partner nearby.

All interviews were audio-recorded using a digital voice recorder after the participant had given their oral informed consent. Only contextual notes from the interview were recorded in a field notebook during and after the interview and were later typed into the corresponding transcribed interview.

### Data management and analysis

All audio-recorded interviews were fully transcribed by a certified French company. Transcriptions were manually coded using an iterative approach.^35^ The deductive approach followed the main themes of the interview guide (see Supplementary Material). Coding of the interviews identified several elements of violence, which were classified using an inductive approach. Coding was carried out in Excel by the first two authors, who discussed and validated the main elements of reproductive violence identified (inappropriate fatphobic remarks, sexual or physical violence, dehumanising care practices, normative pressure, access difficulties and differential treatment) that are reported here. Analysis followed a comprehensive perspective, considering each experience as it was told and felt, and not as it may have objectively happened. The analysis highlights experiences shared by several participants. In line with the insights of the Chicago School,^§^^36^ more singular experiences, sometimes reported by only one person interviewed, were also considered. However marginal and specific such cases may be, they provide elements that help shed light on the issue. The analysis was performed in French. The quotations used here were translated into English by the authors.

### Ethics approval and consent to participate

Ethics approval was obtained from the French Data Protection Authority (CNIL, Commission nationale de l'Informatique et des Libertés, reference 2218847) and the INED Ethics Committee (notice 29, 30/01/2021).

Before giving written consent to take part in the online survey, people were invited to visit the survey website. This described the research and its objectives, and explained the participants’ rights and how they could exercise them (right of access, opposition, rectification and deletion of their responses). At the end of the questionnaire, respondents were asked if they agreed to be contacted later for a semi-structured interview and if so, to provide their contact details. When contacted to take part in the qualitative survey, participants were invited to confirm or withdraw by e-mail their initial written consent. An e-mail was sent to each participant before the interview, reminding them of their rights and their anonymisation number, which could be used to assert their rights once their identifying data had been definitively deleted. Participants gave their oral informed consent for participation and recording of their voices. Recording was voluntary, and participants could refuse. However, all participants gave their consent. The study design, data collection and analysis were conducted in accordance with the Consolidated Criteria for Reporting Qualitative Research (COREQ) guidelines.^37^

## Results

### Study participants

Of the 300 respondents to the online questionnaire who were contacted, 69 answered and agreed to be interviewed (23%). All the interviewees had undergone MAR outside the French legal framework, either by crossing borders to seek reproductive care, in France by importing sperm from abroad (Denmark) or independently “at home” with a private donor or a friend. Participants’ sociodemographic data at the time of interview are presented in Table 1.

**Table 1.** Sociodemographic characteristics of the 69 respondents at the time of interview (2022–2023)

Pseudonym*	Region	Age	Marital situation^a^	Children	Profession^b^	Countries for MAR	Year of last MAR
Alexandra	Paris region	48	same-sex couple	1	librarian	Belgium, France	2010
Alice	Paris region	42	heterosexual couple	1	researcher	Czech Republic	2021
Alix	Nouvelle-Aquitaine	42	single	1	public service executive	Denmark, France	ongoing
Amanda	Paris region	40	same-sex couple	2	teacher	France, Denmark	2020
Anne	Occitanie	42	same-sex couple	2	project manager	The Netherlands	2016
Assia	Paris region	42	heterosexual couple	0^c^	project manager	France, Denmark	2022
Aurélie	Pays de la Loire	43	single	1	communication manager	Spain	ongoing
Bertille	Hauts-de-France	47	heterosexual couple	1	human resources manager	France, Spain	2020
Bintou	Provence-Alpes-Côte d'Azur	42	single	1	manager	Spain	ongoing
Carmen	Hauts-de-France	35	heterosexual couple	1	public service executive	France, Spain	2021
Caroline	Canada	38	heterosexual couple	2	lawyer	Canada	2021
Céline	Nouvelle-Aquitaine	40	single	2	farmer	Belgium	2021
Céline D.	Paris region	37	same-sex couple	1	researcher	Belgium	2021
Chantal	Auvergne-Rhône-Alpes	42	single	2	health executive	Spain	2022
Charline	Grand Est	39	same-sex couple	1	health executive	Spain	2018
Claire	Paris region	35	same-sex couple	1	executive manager	France	2018
Clarisse	Occitanie	42	single	0	researcher	France, Portugal	ongoing
Clément	Paris region	36	same-sex couple	1	teacher	Mexico	2021
Constance	Nouvelle-Aquitaine	41	same-sex couple	4	influencer	Belgium	2015
Dürdane	Champagne-Ardennes	44	heterosexual couple	2	commercial manager	France, Spain	2019
Elisabeth	Paris region	44	heterosexual couple	1	architect	Spain, France, Ukraine	ongoing
Elise	Paris region	44	heterosexual couple	0^c^	marketing manager	France, Czech Republic	2022
Elodie	French overseas territories	40	same-sex couple	0	midwife	France	ongoing
Emilie	Occitanie	39	same-sex couple	0	engineer	Spain, France	ongoing
Fleur	Occitanie	42	single	1	yoga teacher	Spain	2021
Flora	Provence-Alpes-Côte d'Azur	43	single	0^c^	air traffic controller	Spain	ongoing
Françoise	Paris region	41	heterosexual couple	0	engineer	Spain, France	ongoing
Gaël	Auvergne-Rhône-Alpes	31	same-sex couple (trans man with cis man)	1	nurse	Canada	2018
Gaëlle	Paris region	34	same-sex couple	1	project manager	France	ongoing
Gaëlle 2	Auvergne-Rhône-Alpes	47	same-sex couple	2	dentist	France, Denmark	ongoing
Isabelle	Occitanie	38	heterosexual couple	1	lawyer	Denmark	2021
Jean (& Nicolas)	Paris region	33	same-sex couple	0	public service executive	USA	ongoing
Jeanne	Paris region	40	heterosexual couple	1	social worker	USA	2017
Jennifer	Champagne-Ardennes	33	single	1	teacher	Spain, France	ongoing
Julie	Paris region	49	single	1	jurist	Spain	2019
Juliette	Paris region	42	same-sex couple	2	teacher	Switzerland, Spain	2021
Laura	Paris region	39	same-sex couple	1	teacher	Belgium	ongoing
Lola	Paris region	35	heterosexual couple	1	events manager	France, Spain	2022
Louise	Auvergne-Rhône-Alpes	40	single	1	jurist	The Netherlands	2019
Lucie	Nouvelle-Aquitaine	33	same-sex couple	1	teacher	Spain	ongoing
Luna	Nouvelle-Aquitaine	38	single	0	secretary	France, Spain	ongoing
Maëlle	Auvergne-Rhône-Alpes	35	heterosexual couple	1	architect	France, Belgium, Spain	2021
Magali	Occitanie	42	heterosexual couple	0^c^	association manager	Spain	2022
Marie	Pays de la Loire	43	single	0	team manager	Denmark, France	ongoing
Marie-Clémence	Nouvelle-Aquitaine	35	same-sex couple	2	temporary worker in entertainment industry	Spain	2020
Martine	Hauts-de-France	35	heterosexual couple	1	medical doctor	Belgium	2021
Mélissa	Auvergne-Rhône-Alpes	30	same-sex couple	0	nursery assistant	Spain, Belgium, France	ongoing
Mireilla	Paris region	45	heterosexual couple	2	jurist	France, Spain	2020
Molly	Hauts-de-France	28	heterosexual couple (cis woman with trans man)	1	psychotherapist	France	2022
Nathalie	Paris region	47	single	1	IT consultant	Spain, France	2018
Niza	Paris region	38	single	0	press officer	Portugal	ongoing
Paloma	Auvergne-Rhône-Alpes	41	single	0	nursing assistant	Spain, France	ongoing
Pauline	Normandie	33	heterosexual couple	0	veterinarian	France, Spain	ongoing
Pierre	Paris region	35	same-sex couple	1	librarian	USA	2022
Raphaëlle	Grand Est	45	heterosexual couple	1	management assistant	France, Spain	2021
Sandrine	Bretagne	44	heterosexual couple	2	sales representative	France, Spain	2016
Sara (& Steven)	Grand Est	39	heterosexual couple	0^c^	medical doctor	Spain	2022
Sibylle	Paris region	44	same-sex couple	4	press officer	Belgium	2011
Soizic	Paris region	37	same-sex couple	1	archivist	France, Belgium	ongoing
Stella	Paris region	47	single	0^c^	temporary worker in entertainment industry	Greece	2022
Tanaïs	Provence-Alpes-Côte d'Azur	40	single	0	administrative assistant	Spain, France	ongoing
Tiphaine	Pays de la Loire	42	single	1	project manager	Spain, Denmark	ongoing
Valérie	Occitanie	39	heterosexual couple	1	human resources manager	France, Spain	2021
Véronique	Paris region	46	single	0	teacher	Spain	ongoing
Victoire	Paris region	37	same-sex couple	0	commercial director	France, Spain	ongoing
Virginie	Paris region	45	single	0	commercial assistant	France, Spain	ongoing
Virginie 2	Auvergne-Rhône-Alpes	34	single	0	teacher	France, Spain	ongoing
Viviane	Grand Est	33	single	2	magistrate	France, Luxemburg, Germany	2022
Zoé	Nouvelle-Aquitaine	52	heterosexual couple	2	public service executive	France, Spain	2011

*The pseudonyms were chosen by the respondents themselves (in particular so that they could recognise themselves in communications and publications resulting from the research); this explains why some names appear twice and are therefore numbered or followed by an initial.

^a^ Some women were in a relationship but carried out their parenthood plans and their MAR journey on their own.

^b^ Usual profession, as some people were unemployed at the time or in professional retraining.

^c^ Pregnant at the time of interview.

Most of the 69 respondents were women (n = 66), aged between 28 and 52. They lived in various regions of France, in both urban and rural areas and in the French overseas territories. The majority were in a couple (n = 46): 23 heterosexual couples, 19 female couples and 4 male couples. Some marital configurations had changed between the time when MAR was carried out and the time of the interview (some couples were separated, while single women were now in couples). Most respondents were architects, lawyers, project managers, marketing managers, midwives, veterinary surgeons or sales managers, while some worked as administrative assistants, school teachers, care assistants or nurses (n = 10). The majority had one or more children (n = 35), and 6 were pregnant at the time of the interview. Only 6 respondents had undergone so-called at-home insemination, and 3 of these had subsequently resorted to MAR abroad. The vast majority of respondents had used gamete or embryo donation at least once in their MAR journey (n = 58). Respondents mainly went to Spain (n = 38), Denmark (n = 12) and Belgium (n = 10). Very few had resorted to surrogacy (n = 5). The majority had taken steps toward assisted reproduction before the 2021 law revision (n = 52), and some had continued after the revision (n = 23).

In total, 28 interviewees reported one or more experiences of violence during their MAR journey, whether in France or abroad, before or after revision of the French bioethics law.

### Legal and organisational restrictions in MAR: between disillusionment and injustice

Since the reshaping of the legal framework of MAR in France, single women and female couples can legally access such care. This was welcomed by the majority of interviewees because women in general, and the respondents themselves while their project was still ongoing, no longer had to circumvent the law to conceive a child. However, many still strongly criticised the organisation of MAR care in France. They highlighted the healthcare system’s inability to implement the new legal guidelines. This led to neglected care due to insufficient medical staff and to treatment that potentially differed depending on women's characteristics. According to Raphaëlle:

“*So, it's good, it's good to open things up to patients, but if they're treated the way we [heterosexual couples] were treated because there aren’t enough gynaecologists to manage it all, it becomes a bit like mistreatment, because there aren't enough doctors*.” (Raphaëlle, 45 years, in a couple with a man, parents of a child conceived through MAR in Spain)

Waiting lists for gamete donation appear to be long and French fertility centres to be saturated. Interviewees wondered how the selection criteria were applied and how applications were prioritised. They also denounced an abnormal disparity between centres. A small number of interviewees stated that they had been refused for MAR even though they met the current legal criteria:

“They gave us the right to MAR, but in fact we don't have this right. They select for us. What I also find particularly unfair is the difference between the CECOS** *and the various public hospitals. In other words, everyone does things differently. The law is the law. It's a crime to drive through a red light, whether you're in Nantes, Paris or Nice*^††^.” (Aurélie, 43 years, solo mother of a child conceived through MAR in Spain, undergoing MAR in the same country for a second child at the time of the interview)

In the midst of this general shortage of gamete donation, the situation is even worse for racialised people. Laura explained that she went to Belgium for sperm donation and directed egg donation (i.e. from a donor who was known to her) so that her donor would be black like her. Given the shortage of donors in France, and of racialised donors in particular and given that gamete donation for LGBT couples and direct donation^‡‡^ was prohibited, she had no choice but to cross the border:

“*I also really wanted my child to … have some of my skin colour, since I am Black and my partner is White. And (…) [for] black women or women who are not, just not white, donations are extremely rare*.” (Laura, 39 years, in a couple with a woman, one child conceived through directed egg donation and sperm donation in Belgium before the revision of French law)

Other people, in particular Louise, who undertook a solo MAR journey after the law revision, felt it was unfair to have to go abroad when they were now legally entitled to access to MAR in France:

“*And I think it's unfair that because they [the public authorities] aren’t capable of applying their law, of giving people the opportunity to do things according to their law, in fact, I still have to pay and suffer all that. (…) It’s not normal, I should have the right to … well, to have the same care as women in France. After all, it’s their responsibility that they didn’t anticipate all this*.” (Louise, 40 years, solo mother of a child after MAR in the Netherlands)

### Psychological, economic and physical mistreatment

Lack of empathy and derogatory remarks were commonly reported in interviews. There was also a feeling of dehumanisation in the centres. Raphaëlle explained that she felt like “*a womb on legs, with appointments lasting 10 minutes, half of which was spent talking about paperwork and finance (…). In other words, we were just numbers, with so many patients that appointments were rushed through at breakneck speed*.”

In some centres in public hospitals where gamete donations are carried out, consultations with a psychologist seem to be decisive for continuing the MAR process. Luna, who for economic reasons had to start the procedures again in France after unsuccessful attempts in Spain, went to two centres in different regions:

“*At [name of the French centre], it was much colder and without any empathy or kindness, especially from the psychologist … where I … well, in two hours of appointments, I was told that I appeared to be a woman without any emotion and almost inhuman. That's really what she said to me (…). And the fact that she said that to me disturbed me enormously, because I'm the complete opposite of what she described, in fact. So it was quite violent. And … and she even told me at the end of the interview that, if she were asked for her opinion, she'd give a negative opinion … on my application.*” (Luna, 38 years, undergoing MAR since 2008, first in France and in Spain with her male partner, then alone in Spain and now in France again)

Lack of consent was also reported during gynaecological examination. In Portugal, as part of the check-up before insemination, Niza explained that the examination was carried out without her consent.

“*There’s an examination to check that the tubes aren’t blocked. So I went there because everyone had recommended it, all the women had recommended it: ‘It’s great, it’s really gentle, it’s great’. In the end, it’s vaginal touching without consent. And at the time, I thought: ‘That’s not quite right’. But I was in the middle of my examination, and it was only afterwards that I said to myself: ‘No, this isn’t right at all. But, hey, we’ll take it and move on’.*” (Niza, 38 years, single and childless, undergoing solo MAR in Portugal since the end of 2021)

During her MAR journey, Clarisse was exposed to severe violence. She explained that her French gynaecologist tried to take advantage of the illegality of sperm donation for single women and offered to have sex with her:

“*Because he actually took advantage of my … desperate situation, to make me believe certain things, and in fact to have sex with me, which I didn't have (…). You have to realise that, in fact, the fact that things are forbidden in France, until now they were forbidden, means that there are people who will take advantage of the situation to … under the pretext of helping them … *” (Clarisse, 42 years, single and childless, undergoing MAR in Portugal from the end of 2021).

She reported that before offering his services as a donor, this French gynaecologist behaved in a completely inappropriate and violent manner towards her.

“*The exams were very strange, he complimented me a lot and touched my breasts, whereas afterwards I saw that the doctors never touched my breasts for exams. All I had was a gynaecological examination, and he would always touch my breast to see if everything was OK on that side. But it wasn't a normal touch, in fact (…) as far as the gynaecological examination was concerned. He took an enormous amount of time. He had his hand on my thigh and did all sorts of inappropriate things, always a bit on the edge, in fact, and said inappropriate things too, telling me I was perfect.*” (Clarisse)

Also, before 2021, the legal restriction on MAR access led some French health professionals to take advantage of this situation financially. Sibylle, in a couple with a woman and undergoing MAR in Belgium in 2006, reported that the gynaecologist responsible for her follow-up in France before insemination took extra money from her for each consultation.^§§^

“*Well, I was well looked after, but anyway, he explained to me that as I was a lesbian and it was illegal, I had to get cash out, and I really gave him notes. He charged me I don't know how much for each consultation*.” (Sibylle, 44 years, 4 children conceived through MAR in Belgium before the revision of the law)

### Intersecting discrimination against LGBTQIA+ individuals and solo mothers

The interviews showed that some people were dissuaded in some way from using MAR in France even though they met the legal conditions for access. Dissuasion was mainly based on arguments of age or weight. Limitation of access because of weight is not confined to France. It has also been reported in the context of care provided abroad, as in the case of Louise who was asked to lose weight before starting her MAR treatment in the Netherlands.

Age was also cited as a problematic issue. While reimbursed treatment is available in France up to the age of 43 for egg retrieval and 45 for embryo transfer, many women spoke of age as an obstacle to accessing MAR care in France, although they were aged less than 43.

“*It’s: ‘Yes, Madam, so you’re too fat, you’re going to have to lose weight’. It's like: ‘Ah, you're 41, no, we're not taking you, you're … in fact you're too old, you're already rubbish’. So it’s not put like that, but in fact that's what it means, it's … there you go. Plus the delays*.” (Paloma, 41 years, single and childless, undergoing MAR in Spain after unsuccessful at-home insemination)

Like Paloma, Tanaïs encountered institutional restrictions surrounding gamete and embryo donation in France. She was referred to a larger MAR centre in the south of France after being refused in a smaller one. In the smaller centre, access to the procedure was made conditional on her providing both a sperm and an egg donor, an illegal requirement used to expedite placement on the waiting list. In the larger centre, she was seen by a biologist who issued another refusal, citing her advanced age and the excessively long waiting times for potential care. This decision was accompanied by an explicit recommendation to pursue treatment abroad, set out in a formal letter of denial of medical coverage, without any proposal for referral to another MAR centre in France.

In our study population, these deterrents, based on age or weight, mainly affected women who were undertaking MAR on their own. The (future) solo mothers felt that conceiving “alone” was often seen as an irresponsible choice, even though most women explained that they had been planning and preparing for this project for several years. Marie considered that in the CECOS, “there's a tendency … to judge or to infantilise”. In the same vein, this seems to apply also to asexual women such as Paloma, who reported her experience as a member of an association:

“*We've got all these young girls, even older ones, over 30-35, who are virgins because they're asexual, because they're aromantic, because they have a whole host of reasons of their own (…). I don't know if you can imagine, but we're talking about medical violence. When you have to go for a hysterosalpingogram, if you come across an asshole like I did, well, I can only imagine the distress people go through. These young women are told from the outset: ‘Oh yeah, but for artificial insemination, it's going to be complicated if you're a virgin’. I don't see the connection. And it's all these things (…), medical violence, that’s it, we suffer, we all suffer*.” (Paloma)

In addition to limitation of access, care was described as more complicated for some women. In particular, some MAR centres required at least two psychology consultations for single women (whereas only one is required for heterosexual couples or female couples):

“*For example, at the CECOS in [name of the city], they automatically impose two psychology appointments for single women (…) whereas a homosexual couple has just one appointment, like heterosexual couples (…). We have the impression that we have to justify our desire for a child, when we shouldn't have to, or else at that point everyone else should be asked to justify*.” (Marie, 43 years, single and childless, undergoing MAR in Denmark before the revision of the French law, then in France from 2022)

The gynaecologist made it clear to Molly that she would not be a priority in the MAR centre, given her weight in addition to her young age and the fact that she was in a relationship with a trans man who had not yet transitioned:

“*Except that I’m fat and I’m also a lesbian, so I was quickly made to understand, well my gynaecologist quickly made me understand, that for MAR, I wasn't going to get priority*.” (Molly, 28 years, in a couple with a transgender man, parents of a child conceived through at-home insemination)

### Coping with reproductive violence: avoidance strategies and empowerment

In “this journey strewn with pitfalls” (Paloma), the majority of the interviewees did not react when faced with derogatory comments and abuse. They explained that this absence of reaction was due to fear that their MAR care or their request for a donation would be rejected, for instance. Sara, herself a medical doctor, told of the gynaecological violence she suffered (endo-vaginal examination by a student who did not introduce himself and did not speak to her):

“*Afterwards, it’s things that, as I was saying, we're afraid of, well, we don’t say anything, because we’re afraid that we'll be put at the bottom of the list.*” (Sara, 39 years, in a couple with Steven, undergoing MAR in Spain)

Paloma explained that the power of doctors made it impossible to challenge or react: “*The white coat is the boss, they’re God. You mustn’t say anything and you mustn’t … And that’s terrible. And that’s terrible*” (Paloma).

She explained that “by force of circumstance,” due to her particularly difficult solo journey, she became a feminist:

“*But then, through force of circumstance, yeah, I think you become one. You become a feminist along the way. You become one because there are some pretty revolting things out there.*” (Paloma)

Against a backdrop of unequal relations and knowledge, and even possible abuse of power, the majority of interviewees sought information and acquired a solid knowledge of their body, medical procedures and techniques, as well as administrative procedures. They presented themselves as experts, as a “MAR backpacker” (Aurélie), a “veteran of MAR” (Sibylle). Louise, for her part, recognised that she had become “pretty good at self-diagnosis”:

“*As I spent a year dealing with thyroid problems and polycystic ovary syndrome and losing weight, I actually looked at myself a lot, my body, my cycles, I wrote down a billion things and so on. So I was pretty good at self-diagnosis.*” (Louise)

Acquiring knowledge enabled them to exercise counter-power, to confront the power of health professionals and state agents. Louise continued:

“*Solo mothers-to-be are now ‘pussies who know how to read legifrance.fr’ [website containing all French laws and legal procedures]: they now know their rights and can demand them.*” (Louise)

At some point during their MAR journey, most of the women in the study joined associations dedicated to MAR or parenthood (such as BAMP!, a collective bringing together current and former MAR patients as well as individuals experiencing infertility, or Mam’enSolo, an association for (future) single mothers). They also engaged with general online platforms and dedicated social media networks (Facebook or WhatsApp groups or online association websites) to read personal histories, ask questions and interact with others with similar trajectories. Through this social and online solidarity, they exchanged “top tips” (Paloma), such as the names of gynaecologists who, before the law revision, agreed to transcribe prescriptions in France and who were empathetic, addresses of the least expensive pharmacies and the most efficient MAR procedures according to age or medical situation. Through these associations and networks, Tiphaine discovered “sisterhood.”

“*So I’ve met a great community, with kindness and sisterhood … Now that’s a word I didn’t know, but that I learnt with this association. But really, there’s no judgement. […] So it gives me support on a daily basis. And after that, there’s a lot of sharing of experiences*.” (Tiphaine, 42 years, single, mother of a child conceived through MAR in Spain, undergoing at-home insemination for a second child)

Associations and social networks, therefore, appear to be a kind of protection from violence, because they also inform and raise awareness of potential gynaecological violence that may occur during medical care. Niza wondered what she should do next after several unsuccessful attempts in Portugal, and in particular whether she should pursue her project in France. One of her options was a famous hospital in Paris not far from where she lives, which would make things easier. But in this hospital, a doctor specialised in endometriosis had been accused of sexually abusing his patients.

“*I thought: I’d never go there; I’d never risk coming face to face with people who are going to kill me. In short, I said to myself: I’ll never set foot there, I’ll never take the risk of coming face to face with people who might, I don’t know, molest me. In short, I think the journey is hard enough as it is, I don’t want to … *” (Niza)

Another strategy was avoidance: going abroad as previously mentioned, but also changing to another medical centre in the event of violence or dissatisfaction with the care received. Here, social networks and associations played a crucial role. The women no longer felt alone, but understood and supported.

## Discussion

Based on 69 interviews of people who had used MAR outside the legal and/or medical framework, this study revealed the prevalence of reproductive violence in these MAR journeys on the margins of legality. Violence took many forms: psychological, economic and physical. It was expressed mainly through gynaecological violence, including lack of consent,^38^ fatphobia^29,39^ and absence of reproductive agency.^40^ The findings of this study complement those of other research that revealed the presence of reproductive violence in other areas such as abortion, contraception or childbirth.^4,12^ They provide new insights to assert that reproductive violence is found across all areas of reproductive care, in line with the claim that it is a structural effect that forms part of the continuum of gender violence.^13^

The narratives collected in this study reveal how the legal and institutional organisation of care creates conditions of vulnerability that promote reproductive violence. Despite the 2021 revision of the French law, which expanded access, there had been no corresponding reorganisation of gamete donation at the time of the interviews (June 2022–January 2023). Our findings show how the shortage of gametes paves the way to discrimination. To manage the unprecedented and growing demand for gamete donation, and in the absence of clear government guidelines, each medical centre has developed its own approach to handling the flow of requests. By law, doctors are required to assess the motivation of patients before deciding whether to accept them for treatment. However, the concept of “motivation” is not clearly defined, which leaves room for subjective interpretation. As a result, medical professionals may apply implicit biases,^38^ often masked under the guise of medical reasoning, particularly when it comes to gender.^41,42^ In some centres, priority seems to be given to individuals deemed to be future “good parents,” meaning those whose situation aligns with the dominant procreative norm, such as the heterosexual couple norm.^43^ This has the effect of sidelining sexual minorities^44^ and, more particularly, single people, further entrenching disparities in access to reproductive care. This lack of standardisation and the influence of subjective criteria underline the ongoing challenges in ensuring equitable treatment for all individuals seeking assistance with reproduction.

Faced with reproductive violence, some women described their absence of reaction. This is very common in cases of gender-based violence.^45,46^ The situation can be disorienting, making it difficult to immediately recognise certain behaviours as abusive. Despite these experiences, the interviewees were careful not to portray themselves as victims – a common strategy among marginalised people and victims of gender-based violence such as sexual violence.^47^ Instead, they emphasised their resilience and capacity to find alternative solutions, such as seeking care abroad.

Internet platforms, associations and support groups play a crucial role in providing practical assistance, facilitating the exchange of information and promoting integration into collective networks.^48^ These spaces contribute to the development of strategies for resisting or preventing violence. They also promote a better understanding not only of the current conditions of MAR care, but also of what such care should be according to the law and under the French universal healthcare system. People interviewed who received care in France and abroad could compare their experiences, and this often led to a critical reassessment of the French medical system. Although this system provides health insurance and has been more inclusive since 2021, it is marked by inequalities, discrimination and mistreatment, as shown in our results.

Furthermore, since the late 2010s, issues of violence in healthcare, particularly gynaecological and obstetric violence,^8^ have gained visibility and have been increasingly denounced. This growing awareness is enabling women to identify, name and share their experiences of violence, including in the context of MAR. In the wake of the MeToo movement,^49^ personal and shared experiences have fostered empowerment and the emergence of critical perspectives on the organisation and provision of MAR care in France.

### Strengths and limitations

A strength of this study was its large sample of 69 interviews, capturing the diversity of experiences and pathways in accessing MAR outside the legal framework or beyond France’s borders. However, since the majority of participants were recruited through user associations and only 23% of those who agreed to participate in the qualitative study were interviewed, they may have had specific characteristics. In particular, they were certainly exposed to associative discourse and likely had a greater awareness of their rights compared with individuals who were more isolated throughout their reproductive journey. In addition, they were likely more inclined to adopt strategies for resisting stigma and marginalisation.

Another limitation is that the study was not specifically designed to focus on reproductive violence, so this phenomenon may have been only partially described by the interviewees. However, the significance of the narration of reproductive violence, in the course of interviews that did not specifically target this issue, is a strong indicator of the importance of these forms of violence throughout the MAR journey.

Finally, the shortage of gametes from racialised donors in French gamete banks was only approached in one interview (Laura), even though this is an important topic. Phenotype matching between donors and parents is no longer an obligatory procedure in France. However, donors are scarce, so racialised individuals seeking a donor who may increase the chances of having a child who resembles them face much longer waiting times than white individuals. This disparity further highlights the intersectional nature of reproductive inequalities; access to care is influenced not only by legal eligibility but also by broader systemic issues, including racial and social biases.

## Conclusion

Our findings suggest that social networks and associations play a crucial role in providing information and support to face reproductive violence in the MAR journey. The late 2010s saw a proliferation in France of online forums and groups dedicated to MAR, many of which were created by advocacy organisations. Our findings suggest that these spaces have become key sources of empowerment. They allow individuals to share experiences, access practical advice and collectively challenge a healthcare system perceived as discriminatory. In-person support is now complemented by collective and online support, and in this context, solidarity is probably strengthened, and knowledge is easier to acquire. Empowered by both solidarity and knowledge, individuals are not only better equipped to navigate structural barriers, but also to actively resist stigma and assert their reproductive rights.

## Supplementary Material

Supplemental Material: Interview Guide

## Data Availability

The dataset generated and analysed during the current study is not publicly available in order to maintain confidentiality and reduce risk to the participants. The interview guide is published as supplementary material to this manuscript. The research project was approved by the Commission nationale de l'Informatique et des Libertés (reference 2218847) and by the INED Ethics Committee (notice 29, 30/01/2021).

## Footnotes

* People who are marginalised on the basis of their phenotypic characteristics and/or their geographical, cultural and/or religious background, whether real or perceived.

† Article L2141-10 of Law No. 2021–1017 of 2 August 2021 on bioethics.

‡ Outside-ART stands for outside-assisted reproductive technologies. This project is funded by the French National Research Agency (Agence Nationale de la Recherche) – project ID ANR-21-CE36-0006-01.

§ The Chicago School, to which Howard Becker belonged, refers to a body of social science research conducted from the 1930s onwards on marginalised populations, namely those who did not conform to dominant norms. The assumption underlying this research was that societies produce their own margins and exclusions through mechanisms of domination that define what is socially acceptable or “normal” and, by extension, what is not. It is characterised by symbolic interactionism, an approach that argues the social world cannot be understood without considering social interactions and the meanings actors assign to their actions.

** In France, the CECOS (Centres for the Study and Conservation of Human Eggs and Sperm) are the public hospital MAR services authorised to perform gamete donations.

†† Nantes, Paris and Nice are three large French cities covering France from the west (Nantes) to the east (Nice) and from the south (Nice) nearly to the north (Paris).

‡‡ Directed egg donation is still prohibited in France.

§§ In France, medical consultations are covered by the health insurance system, and only extra fees charged by private practitioners are out of pocket.
