# Supplementary material for: From reproductive violence to empowerment: reconquering power in assisted reproduction – a qualitative study
Source: Sex Reprod Health Matters. 2026 Jul 3;33(1):2694254. doi: 10.1080/26410397.2026.2694254 (PMC13403364; doi:10.1080/26410397.2026.2694254)
Supplement: Supplemental Material: Interview Guide [file ZRHM_A_2694254_SM7078.docx]

**Supplementary Electronic Material**

**Interview Guide (translated into English) - Key Themes to Explore**

*What can you tell us about your MAR journey?*

### **1. The MAR Journey**

- Description of the MAR process: stages, methods, timeline
  - Where, with whom, and when?
- Background or history of the journey
- Role of MAR in life trajectory
- Influence of this process on decisions in life and personal and social development?

### **2. Decisions and Choices**

- Understanding the decision to pursue MAR and the choices involved
  - Reasons for pursuing MAR
  - Technical options chosen, decision process
  - Person involved in making decisions
  - Other alternatives considered or pursued (adoption, childfree life, etc)?

### **3. Gamete Donation and Surrogacy (if applicable)**

- In case of gamete donation and/or surrogacy
  - Criteria and motivations for choosing this path
  - Important factors (anonymity? matching?)?
  - Practical steps and difficulties encountered

### **4. Access to Information**

- Information gathered about MAR, destinations, treatment methods, medical centres
  - Information channels (healthcare professionals? associations? social media? personal networks?)
- Free and informed decision?
- Aware of or connected to any "MAR community"?

### **5. Organising the MAR Process**

- Logistical aspects of the journey
  - Funding sources / strategy and management
  - Time off work or schedule reorganisation?

### **6. Challenges Encountered**

- Challenges faced?
  - Personal? Social? Professional? Medical?
  - Physical and/or emotional?

### **7. Sharing Experiences and Support**

- Experience shared with others?
  - Role of family, friends, partners, colleagues
  - Online forums or support groups
- Any taboos, stigma, solidarity encountered?

### **8. Opinions and Perceptions of MAR**

- Views on the strengths and limitations of the French MAR system
- Comparison between experiences abroad and medical care in France (if applicable)
- Affected by legislative changes?
- Requested changes?

### **9. (At the end of the interview) – Obstetric and Gynaecological Violence**

- Awareness of obstetric and gynaecological violence?
- Existence of such violence in the context of MAR?

### **10. (Closing the interview) – Final Reflections**

- Any important aspects of MAR experience that have not been covered during interview
